# Simple Fabrication Process for 2D ZnO Nanowalls and Their Potential Application as a Methane Sensor

**DOI:** 10.3390/s130303941

**Published:** 2013-03-20

**Authors:** Tse-Pu Chen, Sheng-Po Chang, Fei-Yi Hung, Shoou-Jinn Chang, Zhan-Shuo Hu, Kuan-Jen Chen

**Affiliations:** 1 Institute of Microelectronics and Department of Electrical Engineering, Center for Micro/Nano Science and Technology Advanced Optoelectronic Technology Center, National Cheng Kung University, Tainan 701, Taiwan; E-Mails: arthur4092@gmail.com (T.-P.C.); changsj@mail.ncku.edu.tw (S.-J.C.); hzs0105@gmail.com (Z.-S.H.); 2 Department of Materials Science and Engineering, Institute of Nanotechnology and Microsystems Engineering, Center for Micro/Nano Science and Engineering, National Cheng Kung University, Tainan 701, Taiwan; E-Mail: fyhung@mail.ncku.edu.tw; 3 The Instrument Center, National Cheng Kung University, Tainan 701, Taiwan; E-Mail: kjchen1982@gmail.com

**Keywords:** low temperature, nanowalls, methane sensor, ZnO

## Abstract

Two-dimensional (2D) ZnO nanowalls were prepared on a glass substrate by a low-temperature thermal evaporation method, in which the fabrication process did not use a metal catalyst or the pre-deposition of a ZnO seed layer on the substrate. The nanowalls were characterized for their surface morphology, and the structural and optical properties were investigated using scanning electron microscopy (SEM), X-ray diffraction (XRD), transmission electron microscopy (TEM), and photoluminescence (PL). The fabricated ZnO nanowalls have many advantages, such as low growth temperature and good crystal quality, while being fast, low cost, and easy to fabricate. Methane sensor measurements of the ZnO nanowalls show a high sensitivity to methane gas, and rapid response and recovery times. These unique characteristics are attributed to the high surface-to-volume ratio of the ZnO nanowalls. Thus, the ZnO nanowall methane sensor is a potential gas sensor candidate owing to its good performance.

## 1. Introduction

ZnO, with a bandgap of 3.37 eV, a large exciton binding energy of 60 meV at room temperature, and the richest family of nanostructures among semiconductor materials, has attracted interest for various applications in optoelectronics and piezotronics, gas/chemical sensors, transparent electrodes, and field emission devices [1–5]. Recently, gas sensors have been used in many applications such as the control of industrial processes and detection of toxic environmental pollutants. Methane gas is one of many dangerous gases, as it is highly flammable when mixed with air and may cause explosions. Methane is also an asphyxiant gas because it can replace oxygen in a confined space. In addition, methane is a greenhouse gas and increases ozone pollution, which may be hazardous to the human health. However, it is difficult to detect the presence of methane, as it is a colorless and odorless gas at room temperature. Thus, the development of a methane gas sensor is very important. There are many groups that have investigated the detection of gases using various structures: thin film metal oxide, catalysts, metal-insulator-metal (MIM) structures, nanostructures, and MEMS structures [6–10]. Compared with these structures, two-dimensional (2D) ZnO nanostructures such as nanowalls and nanosheets are advantageous as gas and biological sensors because they have a high surface-to-volume ratio. There are many fabrication methods for ZnO-nanowall structures, such as metalorganic chemical vapor deposition (MOCVD) and pulsed laser deposition (PLD). The first study of ZnO nanowalls was reported by Ng *et al.* [11] that used carbothermal reduction and gold-catalyzed VLS (vapor-liquid-solid) processes for growing vertical ZnO nanowalls on a sapphire substrate. Grabowska *et al.* [12] reported ZnO nanowalls grown on an *a*-plane sapphire using a two-step vapor phase transport method and a gold catalyst. Zhang *et al.* [13] grew high quality ZnO nanowalls by a two-step growth method employing oxygen-plasma-assisted molecular beam epitaxy (MBE). Kim *et al.* [14] showed a vertical honeycomb-like pattern of ZnO nanowall networks grown on a GaN/*c*-Al_2_O_3_ substrate with the help of a Au catalyst. Brewster *et al* [15] reported the growth of ZnO nanowalls on an *a*-plane sapphire substrate coated with a 1-nm-thick Au film at 1000 °C. Until now, only a few papers have reported applications for ZnO nanowall structures. Maeng *et al.* [16] fabricated a heterojunction diode comprising n-type ZnO nanowall networks with a hole-conducting p-type polymer. Lee *et al.* [17] measured the electrical characteristics and fabricated a NO_2_-gas application for ZnO nanowall networks. Israr *et al.* [18] utilized ZnO nanowalls for the fabrication of a potentiometric cholesterol biosensor. However, the aforementioned structures required the use of expensive machines, toxic metalorganic precursors and flammable gases, complex processes, metal catalysts, and high temperature processes and fabrication and are limited to unique and expensive substrates. Therefore, it is beneficial to develop a simple, low-cost, rapid, catalyst-free, non-toxic, and low-temperature process.

In this paper, we report the synthesis of vertically aligned ZnO nanowalls on a glass substrate using thermal evaporation. The surface morphology and structural and optical properties of the nanowalls were investigated using scanning electron microscopy (SEM), X-ray diffraction (XRD), transmission electron microscopy (TEM), and photoluminescence (PL). Our fabricated ZnO nanowall gas sensors showed good sensitivity and a fast response time.

## 2. Experimental Procedure

The ZnO nanowalls were synthesized on a glass substrate in a horizontal tube furnace by a simple vapor-phase transport process. Briefly, glass substrates were first cut into multiple 1 × 1 cm dies; then ultrasonically cleaned with acetone, isopropyl alcohol, and deionized water for 10 min; and finally blown dry with clean nitrogen gas. The Zn-powder source material was placed in an alumina boat to serve as a source for precursor vapors that react to form ZnO nanowalls by the vapor-solid (VS) mechanism. A clean glass substrate was placed beside the Zn powder at a distance of approximately 1 cm between the substrate and powder, and the alumina boat was placed at the middle of the furnace. During synthesis, a gas mixture of oxygen and nitrogen were introduced at constant flow rate of 10 and 200 sccm, respectively, and the furnace working pressure was maintained at approximately 20 torr by a rotary pump. The temperature of the furnace was increased to 450 °C and maintained for 60 min. Afterwards, the sample was cooled down to room temperature naturally, and a white coating was observed on the substrate. No metal catalysts and additives were used in this experiment. To fabricate the gas sensor, Pd interdigitated contact electrodes were deposited onto the sample by e-beam evaporation with the use of a metal mask. The area covered by the electrodes was 2 mm wide and 2.2 mm long, the finger spacing was 0.2 mm, and the finger width was 0.1 mm.

The surface morphology and microstructure of the prepared ZnO nanowalls were characterized by field-emission SEM (JSM-7000F) and TEM (JEM-2010). Energy dispersive X-ray spectroscopy (EDS) was also utilized to identify the composition during TEM observation. The crystal structure properties of the ZnO nanowalls were analyzed using XRD. The optical properties of the ZnO nanowalls were investigated by PL at room temperature with a 325 nm He-Cd laser as the excitation source. To measure the gas-sensing properties of the ZnO nanowalls, the sample was placed in a sealed chamber, and CH_4_ gas was injected into the chamber.

## 3. Results and Discussion

**Figure 1.**
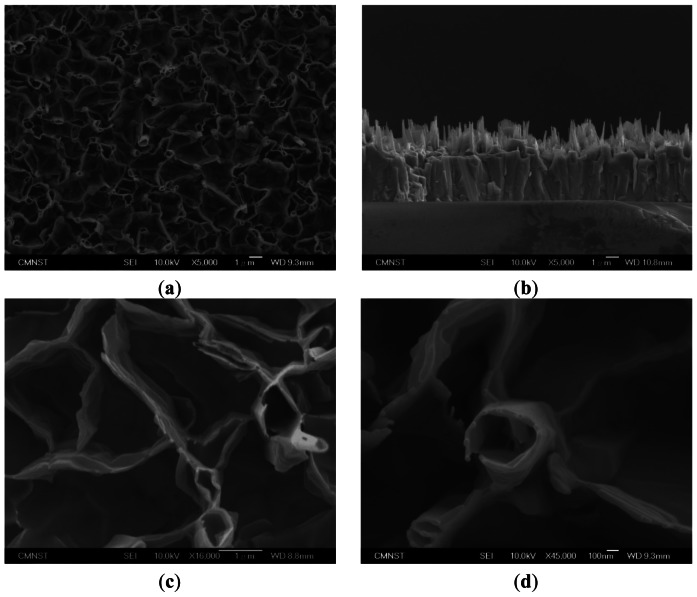
SEM images of ZnO nanowalls grown on a glass substrate by thermal evaporation: (**a**) top view, (**b**) cross-section, and high-magnification of the (**c**) nanowall and (**d**) tube-like structure.

Figure 1(a) shows a top-view SEM image of the ZnO nanowalls. In Figure 1(a), no particles are observed on the top of the ZnO nanowalls, meaning that the ZnO nanowalls grew on the glass substrate by the VS growth mechanism. In other words, the synthesis of ZnO nanowalls only used the Zn powder as the source material in this experiment. The ZnO nanowalls presented a preferred orientation perpendicular to the surface of the substrate, although a few formed at a small angle to the substrate. In Figure 1(b), we see that the ZnO nanowalls are perpendicular to the substrate. However, the ZnO nanowalls were not directly grown on the glass substrate, as there was an additional thick ZnO layer formed between them. The reason for the nanowalls growth is temperature exceeding the zinc melting point of 420 °C, which resulted in the zinc powder melting and evaporation. Oxidation of zinc vapor with oxygen available in the furnace converted it to vapors of zinc oxide. As glass and ZnO lattices are very different, glass substrate being amorphous and ZnO having hexagonal structure, ZnO buffer layer must be formed on the glass substrate. Firstly, the ZnO vapor would form a lot of small ZnO grains on the surface of glass substrate. As the growth proceeds, the ZnO grains become bigger and form tiny nanorods along the c-axis. Then, the ZnO grains grow to merge together, and the tiny nanorods on the surface of the grains also connect with each other. At this stage, the ZnO grains become a buffer layer on the glass substrate and the prototype of the nanowalls has been formed. Finally, a 2D ZnO nanowall structure appeared. In addition to nanowalls, there were some tube-like structures formed with interconnected nanowalls, as seen in Figure 1(c,d). The ZnO nanowalls were approximately 1.3 μm in length and approximately 60 nm in thickness.

**Figure 2.**
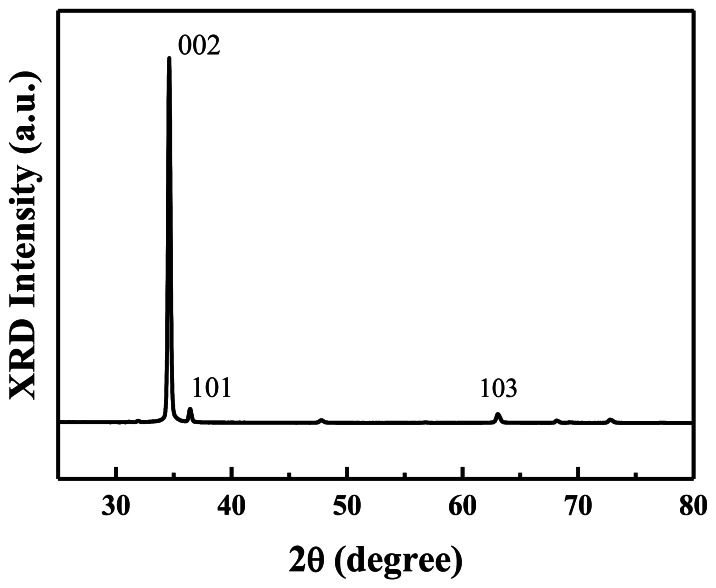
XRD spectrum of the nanowall structure.

Figure 2 shows the XRD spectrum of the prepared ZnO nanowalls. All the diffraction peaks are indexed as a hexagonal wurtzite ZnO structure. A prominent (0002) growth direction indicates that the ZnO nanowalls preferentially grow along the *c*-axis orientation on the substrate. A weak (101) peak is observed in the figure that originates from a few *c*-axis oriented ZnO nanowalls that grew at a small angle to the substrate, as indicated by the (101) peak in the XRD spectrum. This result indicates that well-crystallized ZnO nanowalls with *c*-axis orientation were successfully grown on a glass substrate by a furnace without any metal catalysts or additives.

**Figure 3.**
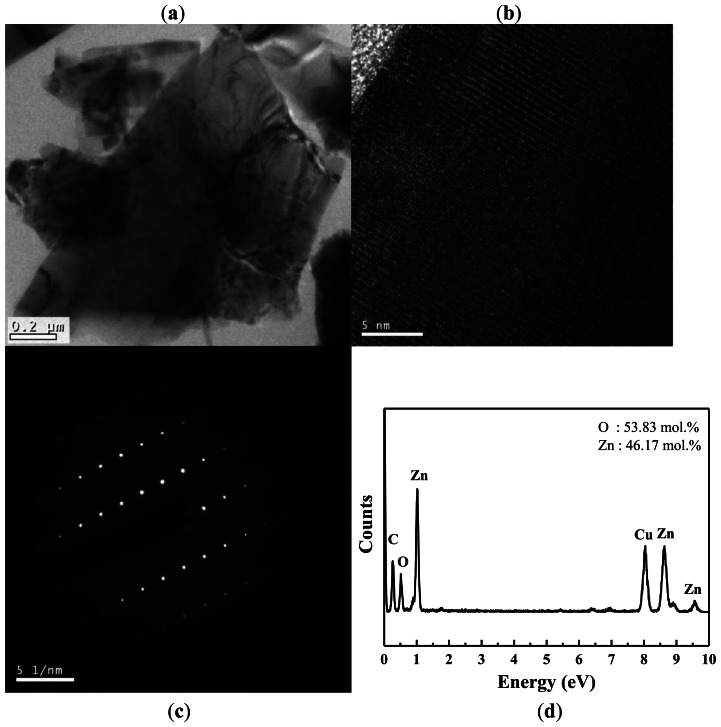
(**a**) Low-magnification TEM image of a nanowall. (**b**) High-resolution TEM image taken from a single ZnO nanowall. (**c**) SAED pattern taken along the (0001) axis of orientation. (**d**) EDS analysis of the ZnO nanowalls.

TEM and EDS were used to further investigate the crystallography and chemical composition of the ZnO nanowalls. Figure 3(a) shows a typical low-magnification TEM image of the ZnO nanowall. The high-resolution TEM (HRTEM) image shown in Figure 3(b) reveals that the ZnO nanowalls have high crystallinity, and the lattice spacing of 0.52 nm corresponds to the d-spacing of the (001) crystal plane of ZnO, implying that they grow along the (001) direction. Figure 3(c) shows the selected-area electron diffraction (SAED) pattern of a ZnO nanowall, indicating that our ZnO nanowalls grew along a *c*-axis orientation as a single crystal with a hexagonal wurtzite structure. These results from TEM and XRD demonstrate that the ZnO nanowalls grown on the glass substrate were preferentially oriented in the (001) direction. The elemental analysis of the ZnO nanowalls by EDS is shown in Figure 3(d). The EDS spectrum shows the existence of Zn, O, C, and Cu. The elements of C and Cu are from the carbon-covered Cu TEM grid, and the elements of Zn and O are from the ZnO nanowalls.

**Figure 4.**
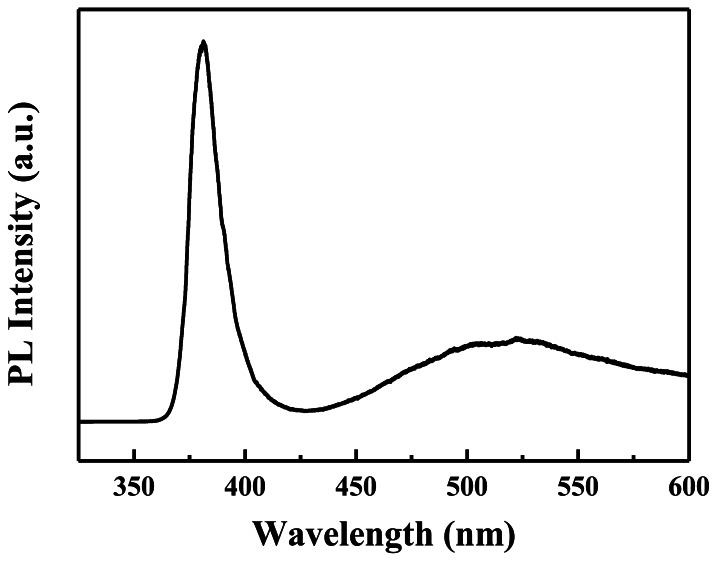
Room temperature PL spectrum of the ZnO nanowalls.

Figure 4 shows the PL spectrum of the ZnO nanowalls for evaluating the optical properties, in which the nanowalls were excited with a 325 nm He-Cd laser at room temperature. Two emission bands are observed: the first is a strong ultraviolet (UV) band, and the other is a weak visible (green/yellow) emission. The UV emission located at 378 nm is assigned to the near-band-edge (NBE) emission, and the visible emission located at 550 nm is related to a deep level or trap state in the ZnO nanowalls, such as interstitial zinc and oxygen vacancies that act as donors at energy levels located below the conduction band edge [19]. Here, a strong UV exciton emission and weaker defect emission prove that the ZnO nanowalls are of good crystal quality.

We then fabricated methane sensors using the furnace-grown ZnO nanowalls. Pd contacts are employed, as Pd is a good catalyst for methane sensing. The I-V characteristics of the nanowall sensor for different concentrations of methane (100 ppm, 500 ppm, 1000 ppm, and 3000 ppm) were recorded in the temperature range of 100–400 °C at a constant bias voltage of 3 V. At room temperature, the current showed a rectifying and symmetrical behavior because of the Pd electrode forming a Schottky barrier on the ZnO surface. The current of the ZnO nanowalls methane sensor was about 3.58 × 10^−6^. A with an applied bias of 3 V. The relative sensitivity ratio, *S*, is expressed in terms of the sensor current in methane gas and in air at a constant voltage:

(1) $$S={\left(\frac{I_{g}-I_{a}}{I_{a}}\right)}_{V}$$

Where *I_g_* is the current in the methane gas, and *I_a_* is the current in air.

**Figure 5.**
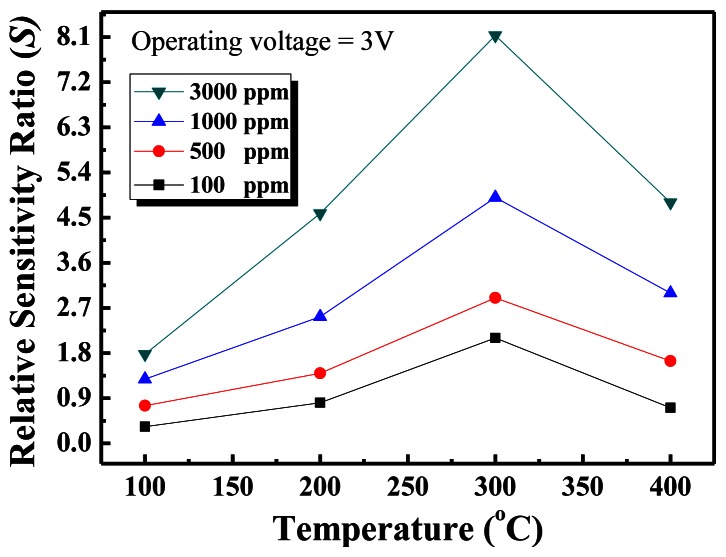
Relative sensitivity ratio *versus* temperature curves of the ZnO nanowall methane sensor.

Figure 5 shows the relative sensitivity ratio *versus* temperature curves of the nanowall sensors for four different methane concentrations. As seen in the figure, the temperature with the maximum response was obtained at 300 °C, and there is an increasing trend in the response as the methane concentration increases, but the response does not reach saturation.

**Figure 6.**
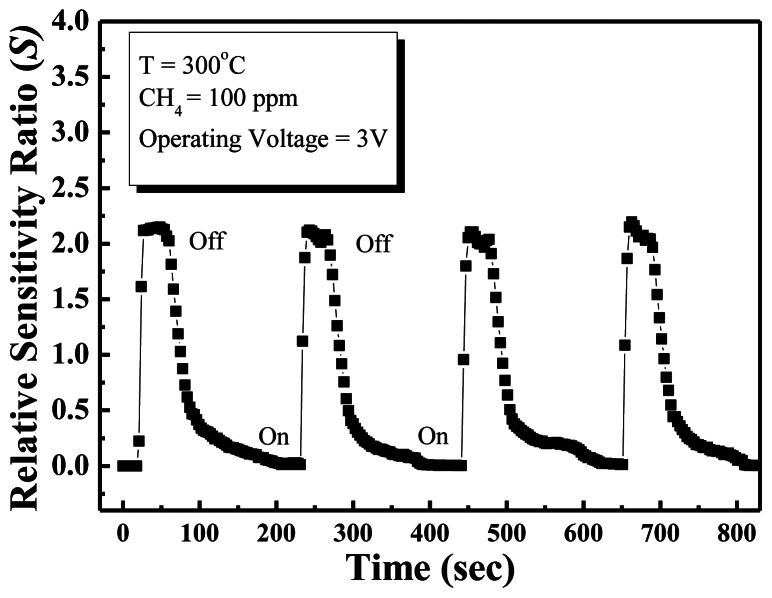
Transient response curves of the ZnO nanowalls at 300 °C and 3 V applied bias.

The response time of the methane sensor is a very important parameter for commercial applications. Figure 6 shows reproducible results for repeated cycles in 100 ppm methane at 300 °C. The response time is defined as the time to reach 67% of the saturation value of the saturation response, and the recovery time is defined as the time to reduce the saturation current to 67%. According to these definitions, the response and recovery times obtained at an operating temperature of 300 °C in the presence of 100 ppm methane gas were approximately 6 s and 21 s, respectively. The ZnO nanowall methane sensor has good detection ability, and quick response and good recovery times. In this experiment, the response and recovery times of the methane sensor are shorter than other conventional methane gas sensor [20,21], which is due to the ZnO nanowalls having a large surface-to-volume ratio and the surfaces of the nanowalls prepared by furnace having good crystal quality.

The conductance of the ZnO nanowall surface is important for methane gas sensing. The detailed mechanism is discussed as follows. ZnO is typically an n-type material owing to the presence of oxygen vacancy defects, which helps oxygen molecules adsorb on the ZnO surface and form negatively charged species by capturing free electrons. These oxygen molecules strongly depend on temperature. The interaction process of the oxygen adsorbed on the surface can be described as follows [22,23]:

(2) $$O_{2(g)}↔O_{2(\text{ads})},$$

(3) $$O_{2(\text{ads})}+e^{-}↔{O_{2(\text{ads})}}^{-}\;\text{for T}<100{}^{\circ}\text{C},$$

(4) $${O_{2(\text{ads})}}^{-}+e^{-}↔2{O_{\left(\text{ads}\right)}}^{-}\;\text{for T}=100-300{}^{\circ}\text{C},\text{and}$$

(5) $${O_{\left(\text{ads}\right)}}^{-}+e^{-}↔{O_{\left(\text{ads}\right)}}^{2-}\;\text{for T}>300{}^{\circ}\text{C}.$$

when the oxygen molecules adsorb on the ZnO surface, a depletion layer is formed nearby and can decrease the carrier concentration. When methane gas is introduced into the chamber, the gas molecules dissociate into a methyl group and hydrogen on the Pd surface and then combine with chemisorbed ionic oxygen molecules on the surface to produce CO_2_, H_2_O, and free electrons. Releasing more free electrons yields more current through the electrodes owing to the increase in conductivity and decrease in the width of the depletion layer, as in the following process [24]:

(6) $$\begin{array}{l} CH_{4}\to CH_{3(\text{ads})}+H_{\left(\text{ads}\right)},\\ CH_{3}+H+4O^{-}\to CO_{2}+2H_{2}O+4e^{-},\text{and}\\ CH_{4}+4O^{-}\to CO_{2}+2H_{2}O+4e^{-}. \end{array}$$

Therefore, we successfully synthesized ZnO nanowalls with a simple, rapid, catalyst-free, and low-temperature process using a tube furnace, and the ZnO nanowall methane sensor has good sensitivity, and fast response and recovery times in methane gas.

## 4. Conclusions

In summary, a simple method for growing well-aligned 2D ZnO nanowalls on glass substrates at a low temperature of 450 °C by thermal evaporation using metallic Zn powder has been reported. This process does not require a metal catalyst or a ZnO seed layer grown on a substrate. The ZnO nanowalls have a wall thickness of 60 nm and an average height of approximately 1.3 μm. For methane sensing, maximum sensitivity was detected at 300 °C. The sensor showed good sensitivity, and rapid response and recovery times in methane gas, attributed to the ZnO nanowalls have a large surface-to-volume ratio that can adsorb more methane gas molecules. Consequently, the ZnO nanowalls are a good candidate for the detection of methane in a dangerous environment.
